# Effectiveness of Learning Systems from Common Image File Types to Detect Osteosarcoma Based on Convolutional Neural Networks (CNNs) Models

**DOI:** 10.3390/jimaging8010002

**Published:** 2021-12-27

**Authors:** Chanunya Loraksa, Sirima Mongkolsomlit, Nitikarn Nimsuk, Meenut Uscharapong, Piya Kiatisevi

**Affiliations:** 1 Medical Engineering, Faculty of Engineering, Thammasat University, Pathum Thani 12121, Thailand; nsnitikarn@engr.tu.ac.th; 2 Faculty of Public Health, Thammasat University, Pathum Thani 12121, Thailand; sirima@fph.tu.ac.th; 3 Department of Medical Services, Lerdsin Hospital, Ministry of Public Health in Thailand, Bangkok 10500, Thailand; meenutusc@gmail.com (M.U.); piyamd@hotmail.com (P.K.)

**Keywords:** osteosarcoma, bone cancer, Convolutional Neural Networks, common image file, computer-aided diagnosis

## Abstract

Osteosarcoma is a rare bone cancer which is more common in children than in adults and has a high chance of metastasizing to the patient’s lungs. Due to initiated cases, it is difficult to diagnose and hard to detect the nodule in a lung at the early state. Convolutional Neural Networks (CNNs) are effectively applied for early state detection by considering CT-scanned images. Transferring patients from small hospitals to the cancer specialized hospital, Lerdsin Hospital, poses difficulties in information sharing because of the privacy and safety regulations. CD-ROM media was allowed for transferring patients’ data to Lerdsin Hospital. Digital Imaging and Communications in Medicine (DICOM) files cannot be stored on a CD-ROM. DICOM must be converted into other common image formats, such as BMP, JPG and PNG formats. Quality of images can affect the accuracy of the CNN models. In this research, the effect of different image formats is studied and experimented. Three popular medical CNN models, VGG-16, ResNet-50 and MobileNet-V2, are considered and used for osteosarcoma detection. The positive and negative class images are corrected from Lerdsin Hospital, and 80% of all images are used as a training dataset, while the rest are used to validate the trained models. Limited training images are simulated by reducing images in the training dataset. Each model is trained and validated by three different image formats, resulting in 54 testing cases. F1-Score and accuracy are calculated and compared for the models’ performance. VGG-16 is the most robust of all the formats. PNG format is the most preferred image format, followed by BMP and JPG formats, respectively.

## 1. Introduction

The global bone cancer rate is 0.2% among all types of cancer. Approximately 3600 patients were diagnosed with bone cancer and around 1720 patients passed away in the year 2020 [1]. The most common type of bone cancer is osteosarcoma, which comprises 28% of adult and 56% of adolescent bone cancer found [2]. Tumors can be found when touched and are painful when pressed. Bones can easily break from moving the body during daily routines. One of the issues in the orthopedic nursing is that the prevalence is so low. As the result, doctors are not familiar with this disease and find it difficult to diagnose. Osteosarcoma is a primary bone malignancy with a particularly high incidence rate in children and adolescents relative to other age groups [3,4]. Within one to two years after surgery, osteosarcoma patients often return with a skinny body and dyspnoea because cancer cells have spread to other organs, especially the lungs. Unfortunately, between 50 and 75% of patients with osteosarcoma will present with clinically detectable metastases from bone to lung [4,5,6], a proportion that has increased with sophisticated methods of detection such as computed tomography (CT).

Computer-aided diagnosis (CAD) of osteosarcoma metastasizes is a popular research topic because it can help doctors to detect the nodule in a patient’s lung at the early state. There are several methods that have been proposed in the past few years. The result from the CAD system can separate nodule and non-nodule images out of the large CT-scanned image series from the Digital Imaging and Communications in Medicine (DICOM) format. The most promising machine learning tools are the Convolutional Neural Networks (CNNs), since they are trainable by using labelled data or images which are then fed into the networks to get the output categories. CNNs are the most popular methods for classifying images [7,8]. They can classify images into suitable categories. A CNN extracts features and learns the important features in the Convolution layers, then classifies the output as a positive or negative diagnosis of osteosarcoma metastasizes using the fully connected layers. This learning algorithm, called a supervised learning algorithm, is used to train a CNN’s model to achieve good performance by using quality images in the dataset, such as balanced dataset images or enhanced images. Then, the quantity of the dataset, as well as the suitable pretrained neural network, all have an influence on how well the learning techniques function [9,10,11,12]. The CNN is a trainable machine learning algorithm and can classify with a high accuracy when provided with a good images dataset. In most cases, the dataset consists of huge images to learn, however, since osteosarcoma metastatic disease is very rare disease, the CT-scanned images are limited. This situation is more severe for transferred patients because of the patient privacy law and government policy in Thailand. Only the CT-scanned image file in CD-ROM media can be sent to the new hospital. The transmission of patient data or image files via the internet is prohibited for safety control. Generally, CD-ROM media has a capacity of 703 MB, therefore, the Digital Imaging and Communications in Medicine (DICOM) file from the CT-Scan machine cannot be written onto one CD-ROM. Due to this situation, the source hospital has to convert the DICOM file to another image file format such as JPG, PNG or BMP to reduce the file size before writing onto CD-ROM and transferring to the destination hospital. Then, the quality of image files is changed due to their format and may affect the accuracy of the network that was used for detecting the nodule in the lung.

One popular image file format was raster format, which consisted of grid pixels combined into a big image. When these images were zoomed in, there would be plenty of visible squares. Therefore, raster was a complex image file format that had a smooth color radiant. However, if size of the image was reduced or enlarged, the lines between pixels would start showing. The difference between pixels could be used in image processing to learn how to detect the edge of the object in an image. In addition, there was another type of image, called a vector image, from mathematical calculation. Vector images displayed in geometric shapes that were created by a graphic program. The image file format was defined by a set of mathematical parameters. The examples of vector files included AI and EPS. Presently, this type of image is not applied to medical use due to the huge file size which affected the storage space and delayed the display time, furthermore, the software in each hospital still cannot support this type of image [13,14,15].

The DICOM file does not contain only pixel image data from the CT scan machine, but it contains extra data such as a patient record, pixel density, sliced size, medical protocol, hospital information and machine specification. Moreover, the pixel data in DICOM file are recorded in a long binary format without being separated into an individual CT sliced image, therefore, the pixel data must be extracted, separated and converted into a series of common CT-scanned image files before they can be used in any machine learning algorithms. Due to the conversion, there are a variety of the image file formats with different qualities.

The common image file formats are BMP, JPG and PNG. Therefore, according to the limited images and different file formats, this research will create the CNNs for osteosarcoma metastatic disease detection and investigate the effect of the number of images in the training dataset when using different image formats, as well as evaluate the performance index F1-Score of the trained networks from different image formats with all formats.

Therefore, the aim of this study is to analyze the effectiveness of learning systems from common image file types to detect osteosarcoma based on CNNs’ models to be a guideline for a radiologist to choose a robust model regardless of image format and dataset size.

## 2. Methods

This study design was conducted as an experimental design in Lerdsin Hospital, Department of Medical Services, Ministry of Public Health in Thailand. According to Lerdsin Hospital, there are were total of 202 cases of the osteosarcoma metastatic disease between 2016 and 2021. The total CT-scanned images from all cases are 269,025 images, but only 2212 images were classified as positive for osteosarcoma metastatic disease. The software was written by Python language version 3.7, based on the PyTorch 1.6.0 platform, and run in the Windows 10 operating system with Nvidia CUDA 10.1 library and GTX1070 hardware acceleration GPU.

### 2.1. Dataset Preparation

#### 2.1.1. Data Acquisition

These experiments were approved by the Research Ethics Committee of the Lerdsin Hospital, Department of Medical Services, Ministry of Public Health in Thailand and The Human Research Ethics Committee of Thammasat University (Science), (HREC-TUSc) for consideration. CT-scanned image data were recorded from SIEMENS SOMATOM DEFINITION 64. Specifications: 78 cm Gantry aperture, OIL/AIR, OIL/WATER tube cooling, 100–80 kW output, range: 28–665 mA (optional 800), max load capacity 227 (299 optional), reconstruction matrices 512 × 512. Siemens CARE Dose 4D low dose technology reduces patient radiation by up to 70%.

#### 2.1.2. Ground Truth

The study included 202 patients with osteosarcoma from the Lerdsin Hospital, Department of Medical Services, Ministry of Public Health in Thailand and had 269,025 image files in Digital Imaging and Communications in Medicine (DICOM) format. Patients’ Lung lungs were scanned. There were 2212 image files indicating the presence of an abnormal nodule. To indicate the correct ground truth, one radiologist and two oncology specialists, a total of three individuals, read the CT-scanned image and identified abnormal images separately. If at least two of the three people identified the same abnormal images, then these images are the positive class images for osteosarcoma metastatic disease, which means this abnormality is possibly caused by bone cancer cells that have migrated to the lungs. The DICOM file cannot be directly applied to the CNNs since it contains both image information and other non-image information, such as hospital name and patient name. All DICOM files must be converted to BMP format using the program that comes with the hospital’s CT-scanner as part of the pre-processing step. Then, the Open Computer Vision (OpenCV) Library is used to convert the image files from BMP to JPG and PNG formats, which are the most common picture file formats used in the medical field and in CNNs’ teaching process. There are 269,025 image files extracted from the patients’ DICOMs, and 2212 positive and 2212 negative class image files are used for training the networks in this study.

#### 2.1.3. Datasets

The quantity and shape of the dataset have a big influence on how well machine learning and deep learning approaches work. As a result, in this study, the total 2212 positive and 2212 negative class image files are separated into train images and test images. Eighty percent of the images in this dataset are used to train the model (1769 images), while twenty percent of the data (443 images) is utilized to evaluate the suggested CAD system’s resilience. In order to simulate the limited training images in a dataset situation and in order to evaluate CNNs as a realistic diagnosis and management tool for osteosarcoma metastasizes, the second dataset is generated. The small dataset contains only ten percent of the training images of the original large dataset and equals 177 images for both positive and negative images. The number of images in the small dataset was selected by considering the average of CT-scanned images that contains nodules of one patient in one CT scan to simulate the situation where a hospital has only one patient with an osteosarcoma disease case.

### 2.2. Pretrained Neural Networks

CNNs have been widely utilized to solve many issues in various fields, but their performance in image processing for health applications is outstanding. There is a lot of research that proposes CAD-based detection for the lesion of disease [16,17]. CNNs are currently the most widely used for the computer vision application using the deep learning (DL) approaches. They can also apply new and powerful regularization techniques to a limited training dataset, which is one of the most important factors in their success [17]. VGG architecture is modest and commonly used in the image classification network because it is easy to construct using the repeating structure when it grows in depth [18]. ResNet is also a popular and commonly used CNN that constructs based on a residue deep structure with skip connections [19]. This structure allows information to flow and hop over a set of layers, therefore resolving the problem of vanishing gradients. The vanishing gradients are the big issue for deep networks because it is difficult for deep networks to transmit the error function backwards. The gradients which are used for training weights and biases in the layers become too tiny beyond a certain point, resulting in a network training stop. MobileNet is proposed by Google for low computational devices to be able to use CNNs. This network is most generally used on mobile devices, such as a mobile phone, or on a mini-computer such as Raspberry Pi [20,21]. MobileNet becomes a popular and commonly used CNN due to its performance and small calculation power required.

Generally, the common CNN networks are provided as pretrained neural networks [9,10,11,12]. The pretrained network is the network that comes with all weights and biases in every layer and provides the best output result. The most important characteristics of a pretrained neural network are the accuracy, speed and size. Greater accuracy increases the specificity and sensitivity for detection. According to previous research, the performance of sixteen popular pretrained neural networks are ranked in descending order based on the overall accuracy: ResNet-50—97.18%, DarkNet-53—95.99%, VGG-19—95.35%, DenseNet-201—95.11%, ResNet-18—94.23%, DarkNet-19—94.08%, SqueezeNet—93.84%, ResNet-101—91.43%, GoogLeNet—90.71%, SuffleNet—90.07%, MobileNet-V2—88.07%, Place365-GoogLeNet—85.69%, Inception-V3—80.37%, Xception—74.62%, RasNet-Mobile—73.75% and Inception-ReNet-V28—72.93% [22].

**Figure 1:**
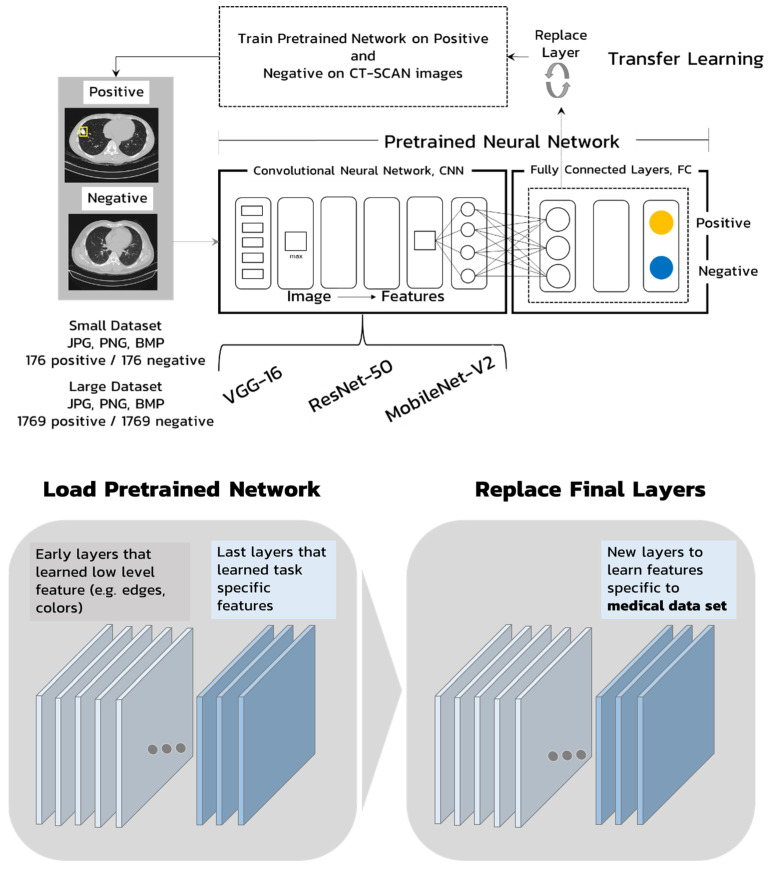
Structure of transfer learning.

In this research, VGG-16, ResNet-50 and MobileNet-V2 CNN models are selected due to the accuracy and simplicity. VGG-16, ResNet-50 and MobileNet-V2 models are the representative of high, medium and low parameter models that were applied and compared in a medical image application [23]. These models are trained for the classification of lesions of osteosarcoma metastasizes from bone to lungs and used to investigate the effect of the different train and test image formats. The pretrained networks are used and modified using transfer learning techniques that replace the fully connected layers into a suitable structure for the osteosarcoma metastasizes’ classification, as shown in Figure 1 [22,24]. There are two classes of output of the networks which are positive and negative classes for nodules.

#### 2.2.1. VGG Model

**Figure 2:**
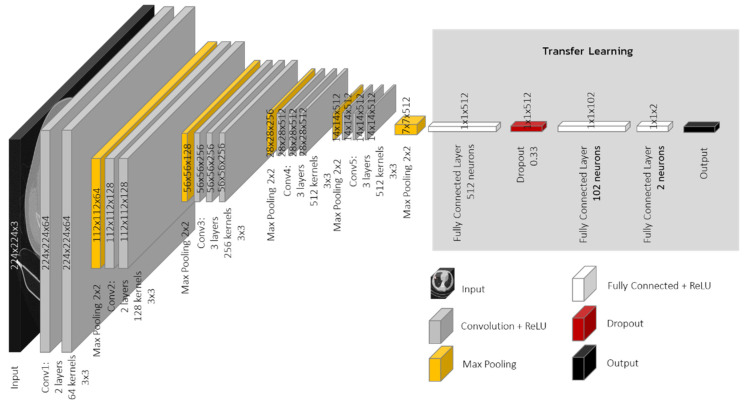
VGG-16 Architecture of this research.

VGG is the CNNs’ deep network proposed to solve image classification problems [18,25]. This network demonstrated that, by increasing the depth from 16 to 19 levels of layers, it can produce a substantially good result. The convolutional layer receives a fixed-size 224 × 224 pixels picture as an input. The picture is processed through a stack of convolutional layers with ReLU activations and filters with extremely tiny receptive fields. The stride of the convolution is likewise set to 1. Spatial pooling is performed after part of the convolutional layers by five max-pooling layers. The advantage of VGG is that, by stacking several convolutional layers with small-sized kernels, the network’s effective receptive field is enhanced while the number of parameters is reduced, as compared to utilizing fewer convolutional layers with bigger kernels for the same receptive field. The authors experimented with a variety of combinations of different depth (9, 11, 16 and 19 layers) [18,25]. According to the original article, the greatest results were obtained for depths ranging from 16 to 19. The pretrained VGG is modified to fit to our research by replacing the original fully connected layers with new fully connected layers that have two output classes (positive and negative classes) and one dropout layer. The dropout layer is added to prevent the overfit issue [26]. Figure 2 shows the VGG-16 architecture of this research. Normally, the convolutional layers in the pretrained CNNs are frozen but, in our case, the CT-scanned training images are represented in a gray scale format. The pretrained model is trained by RGB format images, and they are different from the CT-scanned gray scale format that was used in this research. Therefore, these convolutional layers are not frozen in this research to allow weights and biases in these layers to adapt themselves for a better result.

#### 2.2.2. Residual Networks Model

**Figure 3:**
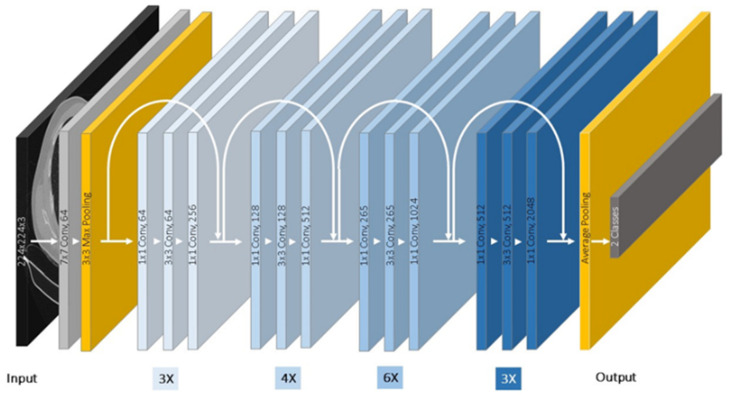
ResNet-50 Architecture of this research.

Residual networks (ResNets) [19] are made up of reformed convolutional layers that learn residual functions based on the input images. According to the original article, this network is easy to optimize and can have a much greater depth. The unique structure of ResNets is a “residual block”, as described in [19,27]. It creates a simple “shortcut connection” for every few convolutional layer that runs parallel to these layers and performs the identity mapping enhancement. Aside from the usage of shortcut links, the network architecture is mostly influenced by the VGG network concept. Small kernels of size 3 × 3 are used in all convolutional layers, which follow two basic design rules. First, the layers have the same number of filters for the same output feature map size. Second, when the feature map size is half (with stride 2 convolutional layers), the number of filters is doubled to maintain the time complexity per layer [25]. ResNet-50′s core principle is to utilize identity mapping to anticipate the need to obtain the final prediction of the preceding layer outputs. ResNet-50 reduces the vanishing gradient effect by using a different shortcut path to circumvent it. The identity mapping enables the model to bypass the superfluous layers. This assists the model in overcoming the overfitting issue to the training set [27]. In this research, the pretrained ResNet-50 CNN model is selected [24,27]. Like the VGG model, the fully connected layers are replaced based on the transfer learning technique by the desired fully connect layer that has two outputs classed with a dropout layer, as shown in Figure 3. The convolutional layers are not frozen as they are in the VGG network.

#### 2.2.3. MobileNet Model

**Figure 4:**
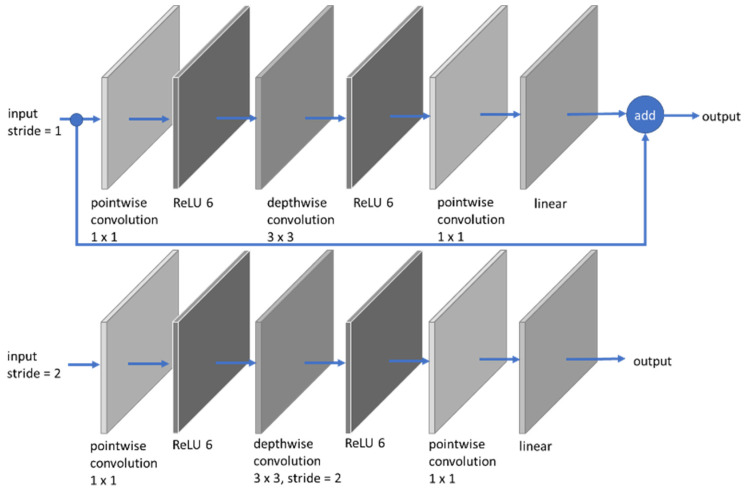
MobileNet-V2 building block architecture of this research.

MobileNets are efficient models for mobile and embedded vision applications, especially for hardware with limited resources. MobileNets are built on a simplified design that uses depthwise separable convolutions to construct low weight deep neural networks instead of regular convolutions, except for the first layer, which is a complete convolution [21]. MobileNet is simpler than VGG-16 and ResNet-50 [8,20,21]. MobileNet-V2 expands on the principles of MobileNet-V1 by employing depthwise separable convolution as efficient building pieces. However, MobileNet-V2 adds two new architectural features [28], which are linear bottlenecks between layers and shortcut connections between bottlenecks. The overview of MobileNet-V2 Architecture is shown in Figure 4, where blocks represent composite convolutional building. In this research, the fully connected layers of MobileNet-V2 are also transferred learning to suit for two classes of output. A dropout layer is also added to the network, similar to VGG-16 and ResNet-50.

### 2.3. Train and Validate the CNNs Networks

There are three possible common CT-scanned image formats that submit to Lerdsin Hospital during the patient transfer procedure, which are BMP, JPG and PNG. In order to obtain the impact from the image file formats and two different size datasets, 18 networks must be created and trained, as shown in Table 1. The CT-scanned small dataset consists of 177 positive and 177 negative class images, each of JPG, PNG and BMP. Training is performed via the three CNN models of VGG-16, ResNet-50 and MobileNet-V2. Thus, there are nine training sets for nine networks (B1 to B9). The CT-scanned large dataset consists of 1769 positive class images and 1769 negative class images, each of JPG, PNG and BMP. Training is conducted via the following three models: VGG-16, ResNet-50 and MobileNet-V2. Thus, we have nine training sets for nine networks (B10 to B18) to ensure that unbiased data lead to proper conclusions [29].

The performance of the trained CNN is evaluated by unseen test images that were separated when the datasets are created. Each trained network is evaluated by three different common image formats to obtain the effect when the training image formats are different from testing image formats. Moreover, in order to make the comparison between networks trained by large and small datasets fair, the same number of unseen test images (443 images) are fed into these networks. For an overview of cases in this research, there are 18 trained networks, and each network is tested with 3 different image formats, thus, there are 54 testing cases, T1 to T54, in total. The evaluation of networks’ performance is performed by calculation of the F1-Score.

### 2.4. Loss Function

The loss function is essential for the neural network training process, in order to modify the weights and biases of a neural network to build a better result. During forward propagation, the neural network is run on training set data and outputs are created that reflect the probability, or confidence in probable labels in the case of classification. These probabilities are compared to the target labels, and the loss function computes a penalty for any difference between the target label and the neural network outputs. The partial derivative of the loss function is generated for each trainable weight of the neural network during backpropagation [30,31,32]. These partial derivatives are used to modify the weights. Backpropagation iteratively changes the trainable weights of a neural network to generate a model with reduced loss under normal conditions. Thus, the suitable loss function must be selected carefully to suit with the output, since the output of the considered CNNs are positive and negative classes for nodules. As a result, the Binary Cross-Entropy (BCE) loss function is introduced for determining classification goodness-of-fit [23]. The BCE is suitable because it is good for a training network with two possible classes of results (positive or negative). The BCE loss function is suitable for our three selected networks and is given by (1) [30,32].

(1) $$Loss=-\frac{1}{N}\overset{N}{\underset{n=1}{\sum }}\left[y_{n}log{\hat{y}}_{n}+\left(1-y_{n}\right)\log\left(1-{\hat{y}}_{n}\right)\right]$$

where

N number of training examples
${\hat{y}}_{n}$ predicted value
$y_{n}$ expected value

### 2.5. Performance Benchmark Scores

In the engineering field, the accuracy, precision, recall and F1-Score [33] are four important metrics that are typically required for measuring the performance of a neural network model [12]. The accuracy, precision and recall scores can be calculated by using Equations (2)–(4), respectively. The calculations are based on four qualitative values, which are True Positive (TP), True Negative (TN), False Positive (FP) and False Negative (FN). TP is the correctly predicted positive value, which means that the value of the actual class is positive, and the value of the predicted class is also positive. TN is the correctly predicted negative value, which means that the value of the actual class is negative, and the value of the predicted class is also negative. FP and FN are the values that occur when the predicted classes are different from the actual classes. If the predicted class is positive but the actual class is negative, this is the FP. On the other hand, if the predicted class is negative but the actual class is positive, this is the FN.

(2) $$\mathrm{Accuracy}\;\left(\%\right)=\frac{\mathrm{TP}+\mathrm{TN}}{\mathrm{TP}+\mathrm{FP}+\mathrm{FN}+\mathrm{TN}}\times 100\%$$

(3) $$\mathrm{Precision}=\frac{\mathrm{TP}}{\mathrm{TP}+\mathrm{FP}}$$

(4) $$\mathrm{Recall}=\frac{\mathrm{TP}}{\mathrm{TP}+\mathrm{FN}}$$

The F1-Score is a metric for how accurate a model is on a given dataset. It is used to assess binary classification algorithms that categorize examples as either positive or negative. The F1-Score, which is defined as the harmonic mean of the model’s precision and recall, is a technique of combining the model’s precision and recall. F1-Score is commonly used to evaluate binary or multiclass classification models’ performance [9,12,33] because this F1-Score takes both false positives and false negatives into account. Accuracy works best if false positives and false negatives have a similar cost. If the cost of false positives and false negatives is very different, the F1-Score is the better measurement benchmark. The F1-Score is defined by Equation (5).

(5) $$F1\;\mathrm{Score}=2\times \frac{\mathrm{Precision}\;\times \;\mathrm{Recall}}{\mathrm{Precision}+\mathrm{Recall}}$$

For the medical field, other scores are commonly used, because it reflexes the medical diagnose information better than the engineering score. These scores are the sensitivity, specificity, F1-Score and Receiver Operating Characteristic (ROC), because the agreement between a proposed (index) test and a reference standard for diagnosing a target condition is examined in diagnostic accuracy studies. The main method is to look at a set of well-defined patients who are blinded to both the index and reference tests in a sequential order. Diagnostic accuracy refers to the degree of agreement between the index test and the reference standard. The reference standard is shown in Table 2. The percentage of malignant nodules that are accurately diagnosed as metastatic nodules is measured by sensitivity, which is the proportion of real positive samples that are appropriately identified. As a result, it is calculated using Equation (6). The sensitivity and recall values are the same. In this case, TP is the number of nodules successfully detected, and FN represents the number of positive nodules that are undetected. Specificity, on the other hand, assesses the percentage of detected negative samples, in which the percentage of nodules that are not malignant is accurately categorized as non-cancerous. Specificity is calculated by Equation (7). TN is the number of non-cancer patients correctly categorized, and FP denotes the number of non-cancer patients incorrectly diagnosed as cancer. F1-Score, on the other hand, calculates the average score, as already explained in Equation (5) [34,35].

(6) $$\mathrm{Sensitivity}=\frac{\mathrm{TP}}{\mathrm{TP}+\mathrm{FN}}$$

(7) $$\mathrm{Specificity}=\frac{\mathrm{TN}}{\mathrm{TN}+\mathrm{FP}}$$

## 3. Results

In order to obtain the performance impact from different quality image formats and different sizes of training data in datasets, the experiments were conducted. The first experiment was conducted by training all three possible CT-scanned image file formats by three different CNNs, which are VGG-16, ResNet-50 and MobileNet-V2. The dataset in the first experiment, called the large dataset, was randomly selected (80%) from 2212 CT-scanned images and transformed into 1769 CT-scanned images in BMP, JPG and PNG formats. These 1769 CT-scanned images contained nodules and were labeled as the positive class images. The rest of images in CT-scanned images were used for evaluating the trained networks. To balance the positive images in the large dataset, the negative CT-scanned images were also randomly selected from other CT-scanned images which did not contain nodules. There were 3538 total images in the large dataset that were used to train the networks. In this experiment, due to limitation of the hospital’s computer resources, a batch size was set to 16 per 1 iteration, which means that 16 images were feedforwarded into the networks and the error from each image was obtained and summarized before using this error to find the gradient and train the networks via back propagated algorithm to reduce the loss value. To cover all the images in the dataset, one epoch requires 222 iterations. The term “epoch” means training the neural network with all the training data for one cycle. Therefore, if the networks are trained for 400 epochs, it requires 88,800 iterations, and if the networks are trained for 2000 epochs, it requires 444,000 iterations.

**Figure 5:**
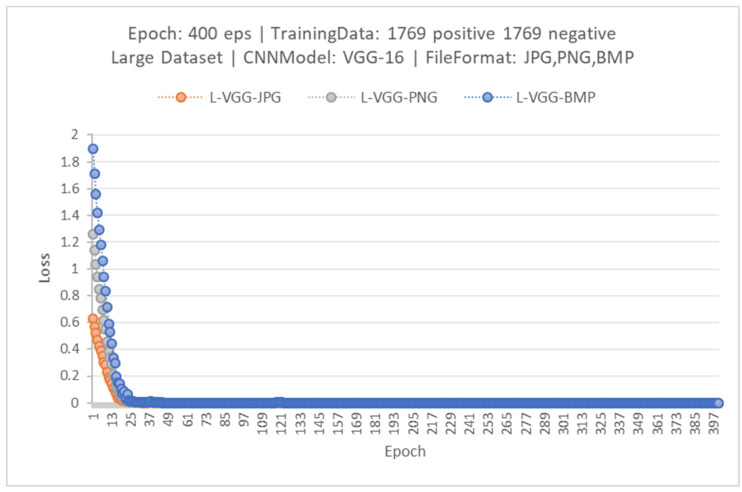
The loss of trained VGG-16 models.

In the first experiment, each model, VGG-16, ResNet-50 and MobileNet-V2, had been trained with three different image file types for 400 epochs. Thus, there were nine trained networks, which were named as L-VGG-JPG, L-VGG-PNG, L-VGG-BMP, L-ResNet-JPG, L-ResNet-PNG, L-ResNet-BMP, L-MobileNet-JPG, L-MobileNet-PNG and L-MobileNet-BMP. These names will be used as the reference in this section. The L is used to explain that the networks had been trained with the large dataset. Figure 5 shows the loss value when training the VGG-16. The loss value graphs of the ResNet-50 and MobileNet show similar behavior to VGG-16, with differences in a training slope and steady-state loss fluctuation value. Loss of the models when trained by different image formats was different at the beginning, however, loss decreased to zero when the number of epochs increased. Loss of VGG-16 (a) was the smallest among the three selected models. By considering loss, the performance of the models when evaluated by the dataset can be obtained. If the loss value is near to zero, there is less error.

**Figure 6:**
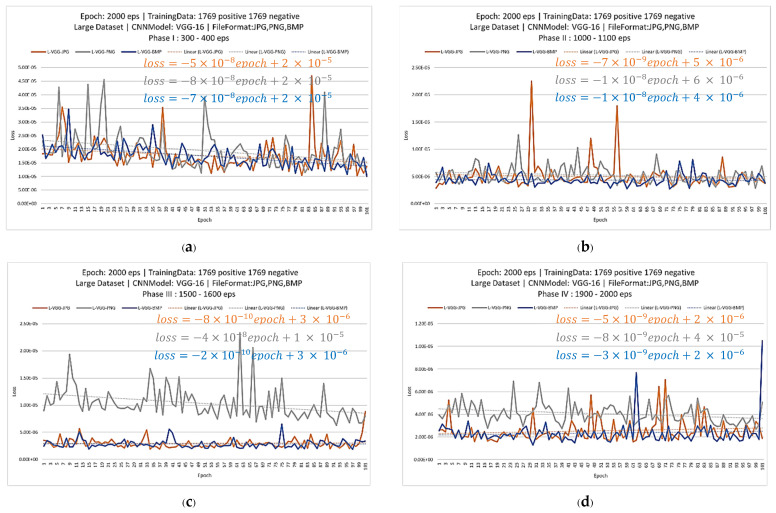
Loss graph of L-VGG-JPG, L-VGG-PNG and L-VGG-BMP phase I-IV (**a**–**d**), respectively.

The one big concern for a radiologist or oncologist who trains the model in his hospital is when he should stop the training. In order to answer this question, the second experiment was conducted. In the second experiment, the CNN models were trained with more epochs in order to obtain the effect of epochs to loss values. Since VGG-16 models have the smallest loss value from the first experiment, only VGG-16 networks were continuously trained up to 2000 epochs. Then, the training graphs were recorded and divided into four training phases. In phase 1, loss values between 300 and 400 epochs were considered, as shown in Figure 6a. Loss values between 1000 and 1100 epochs, 1500 and 1600 epochs and 1900 and 2000 epochs were considered for phase 2, 3 and 4, respectively, as shown in Figure 6b–d. In each phase, the trendlines of losses vs. epochs were calculated and the slopes of the trendlines were collected. Table 3 shows the slope of all training phases.

From Table 3, the slope of each training phase is very close to each other, since the slopes are very small. This second experiment discovered that the loss values converted to zero and the training slopes converged to 0 for all training phases. Thus, it was not necessary to keep training over 400 epochs. Then, the result explains that if the radiologist trains the network and its loss is small enough, he can stop the training process with confidence.

**Figure 7:**
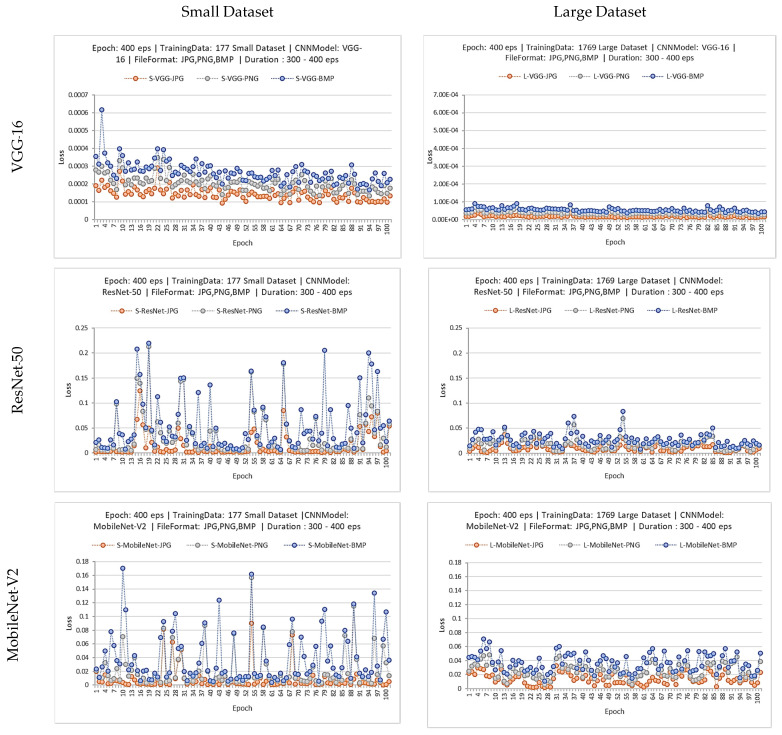
Training graph between 300 to 400 epochs of 18 CNN models trained by the small (**left**) and large (**right**) dataset.

Because osteosarcoma is a very rare bone cancer, it is possible that CT-scanned images in the dataset for training the CNN model may be very small. Therefore, a third experiment was conducted to obtain the impact from the small training dataset to the models’ performances. This experiment aims to simulate the situation for a small hospital that has a very small number of CT-scanned images of patients. To obtain the effect of a small dataset, therefore, 10% of the 2212 positive class images, or 220 images, were randomly picked for model training. The positive 177 images (80% of 220 images) were also randomly selected to be the positive CT-scanned class images for training, combined with 177 negative CT-scanned class images that did not contain nodules, to create the small training dataset. There were 354 total images in the small dataset that were used to train the VGG-16, ResNet-50 and MobileNet-V2. Then, the CNN models were trained for 400 epochs in the same scenario as the first experiment, and there were nine total trained models output from this experiment. The training graphs of the small dataset were displayed and compared with the training graphs of the large dataset. To see this phenomenon, the training graphs were expanded from 300 to 400 epochs, as shown in Figure 7. The fluctuation of VGG-16 model loss is very small when trained by small and large datasets. In the case of ResNet-50 and MobileNet-V2, loss values fluctuated within a larger range if the models were trained by the small dataset. Loss of ResNet-50 fluctuated about 0.23 when trained by the small dataset and 0.07 when trained by the large dataset. Loss of MobileNet-V2 fluctuated about 0.17 when trained by the small dataset and 0.07 when trained by the large dataset. According to the result, VGG-16 is the most robust CNN model when trained by the large and small dataset.

The first, second and third times of experiments show that the losses were approaching zero for all the models for all conditions. However, they are not reflective of real performance when the trained models are used in real situations where the input images are unseen. Therefore, the fourth experiment was conducted to obtain the model’s performance when validated by unseen CT-scanned images. The unseen positive CT-scanned images are the remaining 443 images of each image file type (JPG, PNG and BMP) from the first experiment, which were not included in the large dataset. The unseen 443 negative CT-scanned images were randomly selected from the CT-scanned images that were not included in any dataset and did not have nodules in the images. Then, all 400 epoch-trained models (nine models trained by the small dataset and nine models trained by the large dataset) were evaluated by 3threedifferent unseen image file types, yielding 54 cases in total. The evaluated results and performance scores for the small dataset trained models are shown in Table 4 and the evaluated results and performance scores for the large dataset trained models are shown in Table 5. The experimental result showed that there is a small effect caused by different unseen file formats to the model’s performance. The results shown concluded that the trained models can achieve the same performance when used in the real scenario where the input image formats are different, such as in the case of the transferred patients.

In the case of the small dataset, VGG-16 models have the best F1-Score when evaluated by unseen images, while the accuracy of all models is close to each other. Moreover, VGG-16 model can achieve the same scores and results (TP, TN, FP and FN) regardless of any trained or tested image file formats. Therefore, if there is a limited training CT-scanned image, the VGG-16 model is the best CNN model choice for detecting nodules, since it is the most robust model with the highest F1-Score. In the case of the large dataset, the accuracy of all models decreased, while the F1-Score increased for some models. In the case of ResNet-50, the models that were trained by JPG file format had a higher F1-Score than models that were trained by BMP and PNG formats, respectively. Although MobileNet-V2 models have similar results as the ResNet-50, the models that were trained by BMP format had the highest F1-Score, followed by models that were trained by PNG and JPG formats, respectively. The VGG-16 model was still robust for any trained or tested image formats in the case of the large dataset in consideration of the precision and recall values, even though it did not have the best F1-Score.

To verify the results in the case of training with the large dataset, the 2000 epochs trained models from the fourth experiment were considered. The evaluated results and performance scores for the large dataset 2000 epochs trained models are shown in Table 6. The accuracy and F1-Score of some models decreased and some models increased.

The uncertainty remains for ResNet-50 and MobileNet-V2 in the 2000 epochs training case. For ResNet-50, the model that was trained by JPG format had the highest F1-Score, followed by models that were trained by PNG and BMP formats, respectively. Even though the F1-Score of the ResNet-50 model that was trained by JPG format slightly increased when epochs were increased from 400 to 2000, the F1-Score of the ResNet-50 models that were trained by PNG and BMP formats decreased. The situation was more confused for MobileNet-V2 cases. The MobileNet-V2 model that was trained by BMP format had the highest F1-Score when the model was trained for 400 epochs, but the MobileNet-V2 model that was trained by PNG format had the highest F1-Score when the model was trained for 2000 epochs. The models that were trained by JPG format had a higher F1-Score than the model that was trained by JPG format when epochs were increased to 2000. For VGG-16, all scores remained the same even if the epochs were increased. From this result, the VGG-16 model is still the most robust CNN model for detecting nodules in a CT-scanned image file. It is not easy to find the number of epochs required for the ResNet-50 and MobileNet-V2 models to have the best F1-Score. In addition, the image file type formats affected the score of the ResNet-50 and MobileNet-V2 inconclusively.

All experiments show low F1-Scores and accuracy in all models, regardless of any training or testing image formats, due to small nodule size in the CT-scanned image. However, the experiments discovered the robustness of each model according to different image formats, which addresses the issue of the transferred patients between hospitals in Thailand. In varieties of common image formats that can be sent to Lerdsin Hospital, the experimental results show a guideline for a radiologist to select the VGG-16 model in order to obtain the consistency, accuracy and F1-Scores output regardless of image format and dataset size. Other models show fluctuation and unpredictable F1-Scores and accuracy when these models are trained and tested with different image formats and a different number of epochs. The experiment also provides a guideline for a training where, if the model loss is low enough, then, it is not necessary to keep training the model. Finally, the radiologist can be comfortable using the VGG-16 model for any common image file formats that are already on hand or in the future.

## 4. Conclusions and Discussion

This research aims to analyze the effectiveness of learning systems from common image file types to detect osteosarcoma based on CNNs’ models. There are three popular CT-scanned CNN models investigated in this research, which are VGG-16, ResNet-50 and MobileNet-V2 models. Osteosarcoma bone cancer is a rare type of cancer, thus, it is hard to detect even when advanced computer aided technology, such as machine learning or CNNs, is applied [36]. There are limited cases and resources for successful machine learning. Furthermore, there is an additional problem in Thailand. According to the technical and policy issues in the case of transferred patients in Lerdsin Hospital, all patient records and image files must be written in CD-ROM media for safety purposes. The patient’s DICOM files could not be transferred from the source hospital to the destination hospital because they could not be put onto CD-ROM, therefore, the DICOM files must be transformed into other common CT-scanned image file formats [37,38]. The three common CT-scanned image file formats that are sent to Lerdsin Hospital are BMP, JPG and PNG formats [15,39]. This research searched for the answer to the question of the impact from different file formats on the CNN models while training and on performance validation. This conclusion can leverage the telemedicine operation in Thailand. Hospitals in remote areas can use the online detection system by using any kind of CT-scanned image format, yet achieve the same result. The findings indicated that there are uncertain and inconclusive results for ResNet-50 and MobileNet-V2 when the models were trained by different CT-scanned image formats. Also, the F1-Score of the same model was changed when the epochs were increased. This result implied that ResNet-50 and MobileNet-V2 are not robust for osteosarcoma bone cancer metastasized to lung detection when trained by different training image formats. They are dependent on both training image format and number of epochs, which lead to unclear conclusion about how many training epochs are required in order to obtain the best F1-Score and which training image format should be used. However, in the case of the VGG-16 model, the results from both models trained by large and small datasets were consistent. The different CT-scanned image formats did not affect performance scores of the trained models in any case. VGG-16 is the most robust CNN model for osteosarcoma bone cancer metastasized to lung detection. Due to the experimental results, the file formats have little impact on the overall performance scores. Thus, the criteria for selecting image formats must be the image size and quality which could be most practically used. The average CT-scanned image sizes of the JPG, PNG and BMP formats in the datasets are 177.22, 271.10 and 1029.92 Kilobytes, respectively.

Therefore, the PNG image format is preferred for both training and validating models in medical applications, because the PNG format is a patent-free and lossless compression image format that has been proven as the best quality medical image for radiologic application [15,39] with a small storage size [37] that is capable for a small hospital.

**Figure 8:**
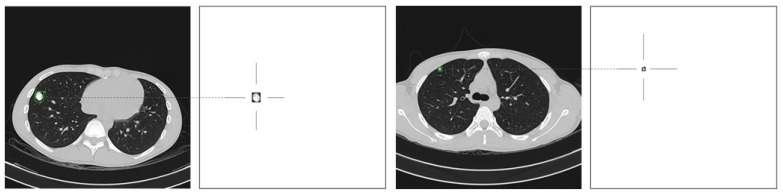
Example of nodule areas compared with image areas.

F1-Score decreased when the training epochs increased. This phenomenon from this research also has been investigated and discussed for further understanding. According to the positive class images, as shown in Figure 8, the area of the nodule in the patient lung is tiny when compared with the whole image area. This situation makes it difficult even for experienced radiologists to detect the positive nodules in the image. Due to this fact, to increase in the performance of the models for detecting the osteosarcoma nodules in the lung, and the more sophisticated machine learning algorithms or CNN models that can identify the region of interest that has the most probability to contain the nodules, must be further investigated, such as a Region-based Convolutional Neural Network (RCNN) or Single Shot Detection (SSD) framework.

## Figures and Tables

**Table 1:** Osteosarcoma nodules that have metastasized from bone to lung datasets for training.

	Codes	Details	Positive (No. of Image)	Negative (No. of Image)	Total (No. of Image)
Small Dataset	B1_VGG16JPG	VGG16 trained on JPG	177	177	354
	B2_VGG16PNG	VGG16 trained on PNG	177	177	354
	B3_VGG16BMP	VGG16 trained on BMP	177	177	354
	B4_ResNetJPG	ResNet50 trained on JPG	177	177	354
	B5_ResNetPNG	ResNet50 trained on PNG	177	177	354
	B6_ResNetBMP	ResNet50 trained on BMP	177	177	354
	B7_MobileNetJPG	MobileNetV2 trained on JPG	177	177	354
	B8_MobileNetPNG	MobileNetV2 trained on PNG	177	177	354
	B9_MobileNetBMP	MobileNetV2 trained on BMP	177	177	354
Large Dataset	B10_VGG16JPG	VGG16 trained on JPG	1769	1769	3565
	B11_VGG16PNG	VGG16 trained on PNG	1769	1769	3565
	B12_VGG16BMP	VGG16 trained on BMP	1769	1769	3565
	B13_ResNetJPG	ResNet50 trained on JPG	1769	1769	3565
	B14_ResNetPNG	ResNet50 trained on PNG	1769	1769	3565
	B15_ResNetBMP	ResNet50 trained on BMP	1769	1769	3565
	B16_MobileNetJPG	MobileNetV2 trained on JPG	1769	1769	3565
	B17_MobileNetPNG	MobileNetV2 trained on PNG	1769	1769	3565
	B18_MobileNetBMP	MobileNetV2 trained on BMP	1769	1769	3565
		Total	17,514	17,514	35,055

**Table 2:** Reference standard.

	Disease Present	Disease Absent	Total
Index Test Positive	True Positive (TP)	False Positive (FP)	TP + FP
Index Test Negative	False Negative (FN)	True Negative (TN)	FN + TN
Total	TP + FN	FP + TN	

**Table 3:** Slopes of trendlines in four training phases.

Period	Trendline Slopes		
	JPG	PNG	BMP
300–400	$-5\times 10^{-8}$	$-8\times 10^{-8}$	$-7\times 10^{-8}$
1000–1100	$-7\times 10^{-9}$	$-1\times 10^{-8}$	$-1\times 10^{-8}$
1500–1600	$-8\times 10^{-10}$	$-4\times 10^{-8}$	$-2\times 10^{-10}$
1900–2000	$-5\times 10^{-9}$	$-8\times 10^{-9}$	$-3\times 10^{-9}$

**Table 4:** Experimental results of small dataset trained 400 epochs models when evaluated by common file formats.

	Format	Format	TP	TN	FP	FN	Accuracy	Precision	Recall	F1 Score
	Trained	Tested								
VGG-16	BMP	BMP	162	355	88	281	0.583	0.648	0.366	0.467
		JPG	161	357	86	282	0.584	0.652	0.363	0.467
		PNG	162	355	88	281	0.583	0.648	0.366	0.467
	JPG	BMP	179	349	94	264	0.596	0.656	0.404	0.500
		JPG	174	348	95	269	0.589	0.647	0.393	0.489
		PNG	179	349	94	264	0.596	0.656	0.404	0.500
	PNG	BMP	161	352	91	282	0.579	0.639	0.363	0.463
		JPG	154	354	89	289	0.573	0.634	0.348	0.449
		PNG	161	352	91	282	0.579	0.639	0.363	0.463
ResNet-50	BMP	BMP	99	394	49	344	0.556	0.669	0.223	0.335
		JPG	100	398	45	343	0.562	0.690	0.225	0.340
		PNG	99	394	49	344	0.556	0.669	0.223	0.335
	JPG	BMP	83	408	35	360	0.554	0.703	0.187	0.296
		JPG	81	409	34	362	0.553	0.704	0.183	0.290
		PNG	83	408	35	360	0.554	0.703	0.187	0.296
	PNG	BMP	87	385	58	356	0.533	0.600	0.196	0.296
		JPG	83	388	55	360	0.532	0.601	0.187	0.286
		PNG	87	385	58	356	0.533	0.600	0.196	0.296
MobileNet-V2	BMP	BMP	86	418	25	357	0.569	0.775	0.194	0.310
		JPG	83	419	24	360	0.566	0.776	0.187	0.301
		PNG	86	418	25	357	0.569	0.775	0.194	0.310
	JPG	BMP	93	428	15	350	0.588	0.861	0.210	0.337
		JPG	84	425	18	359	0.574	0.823	0.190	0.308
		PNG	93	428	15	350	0.588	0.861	0.210	0.337
	PNG	BMP	88	423	20	355	0.577	0.815	0.199	0.319
		JPG	95	424	19	348	0.586	0.833	0.214	0.341
		PNG	88	423	20	355	0.577	0.815	0.199	0.319

**Table 5:** Experimental results of large dataset trained 400 epochs models when evaluated by common file formats.

	Format	Format	TP	TN	FP	FN	Accuracy	Precision	Recall	F1 Score
	Trained	Tested								
VGG-16	BMP	BMP	117	270	173	326	0.437	0.403	0.264	0.319
		JPG	118	269	174	325	0.437	0.404	0.266	0.321
		PNG	117	270	173	326	0.437	0.403	0.264	0.319
	JPG	BMP	116	275	168	327	0.441	0.408	0.262	0.319
		JPG	116	274	169	327	0.440	0.407	0.262	0.319
		PNG	116	275	168	327	0.441	0.408	0.262	0.319
	PNG	BMP	115	272	171	328	0.437	0.402	0.259	0.315
		JPG	115	273	170	328	0.438	0.403	0.259	0.315
		PNG	115	272	171	328	0.437	0.402	0.259	0.315
ResNet-50	BMP	BMP	160	190	253	283	0.395	0.387	0.361	0.374
		JPG	154	193	250	289	0.392	0.381	0.348	0.364
		PNG	160	190	253	283	0.395	0.387	0.361	0.374
	JPG	BMP	171	184	259	272	0.401	0.398	0.386	0.392
		JPG	161	193	250	282	0.399	0.392	0.363	0.377
		PNG	171	184	259	272	0.401	0.398	0.386	0.392
	PNG	BMP	140	250	193	303	0.440	0.420	0.316	0.361
		JPG	130	221	222	313	0.396	0.369	0.293	0.327
		PNG	140	250	193	303	0.440	0.420	0.316	0.361
MobileNet-V2	BMP	BMP	176	201	242	267	0.425	0.421	0.397	0.409
		JPG	187	198	245	256	0.434	0.433	0.422	0.427
		PNG	176	201	242	267	0.425	0.421	0.397	0.409
	JPG	BMP	130	223	220	313	0.398	0.371	0.293	0.328
		JPG	127	227	216	316	0.399	0.370	0.287	0.323
		PNG	130	223	220	313	0.398	0.371	0.293	0.328
	PNG	BMP	140	250	193	303	0.440	0.420	0.316	0.361
		JPG	152	244	199	291	0.447	0.433	0.343	0.383
		PNG	140	250	193	303	0.440	0.420	0.316	0.361

**Table 6:** Experimental results of large dataset trained 2000 epochs models when evaluated by common file formats.

	Format	Format	TP	TN	FP	FN	Accuracy	Precision	Recall	F1 Score
	Trained	Tested								
VGG-16	BMP	BMP	111	292	151	332	0.455	0.424	0.250	0.315
		JPG	113	288	155	330	0.453	0.422	0.255	0.318
		PNG	111	292	151	332	0.455	0.424	0.250	0.315
	JPG	BMP	115	267	176	328	0.431	0.395	0.259	0.313
		JPG	114	273	170	329	0.437	0.401	0.257	0.314
		PNG	115	267	176	328	0.431	0.395	0.259	0.313
	PNG	BMP	112	264	179	331	0.424	0.385	0.253	0.305
		JPG	117	265	178	326	0.431	0.397	0.264	0.317
		PNG	112	264	179	331	0.424	0.385	0.253	0.305
ResNet-50	BMP	BMP	69	289	154	374	0.404	0.309	0.156	0.207
		JPG	64	300	143	379	0.411	0.309	0.144	0.197
		PNG	69	289	154	374	0.404	0.309	0.156	0.207
	JPG	BMP	180	184	259	263	0.411	0.410	0.406	0.408
		JPG	179	184	259	264	0.410	0.409	0.404	0.406
		PNG	180	184	259	263	0.411	0.410	0.406	0.408
	PNG	BMP	106	245	198	337	0.396	0.349	0.239	0.283
		JPG	101	246	197	342	0.392	0.339	0.228	0.272
		PNG	106	245	198	337	0.396	0.349	0.239	0.283
MobileNet-V2	BMP	BMP	140	209	234	303	0.394	0.374	0.316	0.343
		JPG	131	223	220	312	0.399	0.373	0.296	0.330
		PNG	140	209	234	303	0.394	0.374	0.316	0.343
	JPG	BMP	164	225	218	279	0.439	0.429	0.370	0.397
		JPG	164	224	219	279	0.438	0.428	0.370	0.397
		PNG	164	225	218	279	0.439	0.429	0.370	0.397
	PNG	BMP	223	201	242	220	0.478	0.479	0.503	0.491
		JPG	225	205	238	218	0.485	0.486	0.508	0.497
		PNG	223	201	242	220	0.478	0.479	0.503	0.491

## Data Availability

The raw data supporting the conclusions of this manuscript can be made available on request by the authors to any qualified researcher.

## References

[1] Siegel R.L. (2020). Cancer Statistics 2020. CA Cancer J. Clin..

[2] Cancer.Net Doctor-Approved Patient Information from ASCO Publications, Bone Cancer (Sarcoma of Bone): Statistics, Approved by the Cancer.Net Editorial Board, 01/2021. https://www.cancer.net/cancer-types/bone-cancer-sarcoma-bone/statistics.

[3] Martin J.W., Squire J., Zielenska M. (2012). The Genetics of Osteosarcoma. Sarcoma.

[4] Matsubara E., Mori T., Koga T., Shibata H., Ikeda K., Shiraishi K., Suzuki M. (2015). Metastasectomy of Pulmonary Metastases from Osteosarcoma: Prognostic Factors and Indication for Repeat Metastasectomy. J. Respir. Med..

[5] Su W.T., Chewning J., Abramson S., Rosen N., Gholizadeh M., Healey J., Meyers P., La Quaglia M.P. (2004). Surgical management and outcome of osteosarcoma patients with unilateral pulmonary metastases. J. Pediatr. Surg..

[6] Bramwell V. (1997). Metastatic Osteosarcoma: A Review of Current Issues in Systemic Treatment. Sarcoma.

[7] Liang X., Yu J., Liao J., Chen Z. (2020). Convolutional Neural Network for Breast and Thyroid Nodules Diagnosis in Ultrasound Imaging. BioMed Res. Int..

[8] Lin Z., Ye H., Zhan B., Huang X. (2020). An Efficient Network for Surface Defect Detection. Appl. Sci..

[9] Narin A., Kaya C., Pamuk Z. (2021). Automatic detection of coronavirus disease (COVID-19) using X-ray images and deep convolutional neural networks. Pattern Anal. Appl..

[10] Sethy P.K., Behera S.K., Ratha P.K., Biswas P. (2020). Detection of coronavirus Disease (COVID-19) based on Deep Features and Support Vector Machine. Int. J. Math. Eng. Manag. Sci..

[11] Zhang J., Xie Y., Pang G., Liao Z., Verjans J., Li W., Sun Z., He J., Li Y., Shen C. (2021). Viral Pneumonia Screening on Chest X-rays Using Confidence-Aware Anomaly Detection. IEEE Trans. Med. Imaging.

[12] Hemdan E.E.-D., Shouman M.A., Karar M.E. COVIDX-Net: A Framework of Deep Learning Classifiers to Diagnose COVID-19 in X-ray Images. https://arxiv.org/abs/2003.11055.

[13] Sakshica D., Gupta K. (2015). Various Raster and Vector Image File Formats. Int. J. Adv. Res. Comput. Commun. Eng..

[14] Gonzalez R.C., Woods R.E. (2008). Digital Image Processing.

[15] Tan L.K. (2006). Image file formats. Biomed. Imaging Interv. J..

[16] Naseer A., Yasir T., Azhar A., Shakeel T., Zafar K. (2021). Computer-Aided Brain Tumor Diagnosis: Performance Evaluation of Deep Learner CNN Using Augmented Brain MRI. Int. J. Biomed. Imaging.

[17] Signoroni A., Savardi M., Baronio A., Benini S. (2019). Deep Learning Meets Hyperspectral Image Analysis: A Multidisciplinary Review. J. Imaging.

[18] Simonyan K., Zisserman A. (2014). Very Deep Convolutional Networks for Large-Scale Image Recognition. arXiv.

[19] He K., Zhang X., Ren S., Sun J. Deep Residual Learning for Image Recognition. Proceedings of the IEEE Conference on Computer Vision and Pattern Recognition.

[20] Sandler M., Howard A., Zhu M., Zhmoginov A., Chen L. MobileNetV2: Inverted residuals and linear bottlenecks. Proceedings of the IEEE/CVF Conference on Computer Vision and Pattern Recognition (CVPR).

[21] Howard A.G., Zhu M., Chen B., Kalenichenko D., Wang W., Weyand T., Andreetto M., Adam H. MobileNets: Efficient Convolutional Neural Networks for Mobile Vision Applications. https://arxiv.org/pdf/1704.04861.pdf.

[22] Elgendi M., Fletcher R., Howard N., Menon C., Ward R. (2020). The Evaluation of Deep Neural Networks and X-Ray as a Practical Alternative for Diagnosis and Management of COVID-19. medRxiv.

[23] Zulkifley M.A., Abdani S.R., Zulkifley N.H. (2020). Automated Bone Age Assessment with Image Registration Using Hand X-ray Images. Appl. Sci..

[24] Peng J., Kang S., Ning Z., Deng H., Shen J., Xu Y., Zhang J., Zhao W., Li X., Gong W. (2020). Residual convolutional neural network for predicting response of transarterial chemoembolization in hepatocellular carcinoma from CT imaging. Eur. Radiol..

[25] Tsochatzidis L., Costaridou L., Pratikakis I. (2019). Deep Learning for Breast Cancer Diagnosis from Mammograms—A Comparative Study. J. Imaging.

[26] Krizhevsky A., Sutskever I., Hinton G.E. (2017). ImageNet classification with deep convolutional neural networks. Commun. ACM.

[27] ElGhany S.A., Ibraheem M.R., Alruwaili M., Elmogy M. (2021). Diagnosis of Various Skin Cancer Lesions Based on Fine-Tuned ResNet50 Deep Network. Comput. Mater. Contin..

[28] (2018). Google AI Blog The latest from Google Research, Mark Sandler, Andrew Howard, MobileNetV2: The Next Generation of On-Device Computer Vision Networks. https://ai.googleblog.com/2018/04/mobilenetv2-next-generation-of-on.html#1.

[29] Šimundić A.-M. (2013). Bias in research. Biochem. Med..

[30] Ho Y., Wookey S. (2020). The Real-World-Weight Cross-Entropy Loss Function: Modeling the Costs of Mislabeling. IEEE Access.

[31] Dufourq E., Bassett B.A. Automated Problem Identification: Regression vs. Classification via Evolutionary Deep Networks. Proceedings of the South African Institute of Computer Scientists and Information Technologists.

[32] Zhang Z., Sabuncu M.R. Generalized Cross Entropy Loss for Training Deep Neural Networks with Noisy Labels. Proceedings of the 32nd Conference on Neural Information Processing Systems, NeurIPS.

[33] Sasaki Y. (2007). The Truth of the F-Measure. https://www.toyota-ti.ac.jp/Lab/Denshi/COIN/people/yutaka.sasaki/F-measure-YS-26Oct07.pdf.

[34] Chen H.-C., Sunardi, Liau B.-Y., Lin C.-Y., Akbari V.B.H., Lung C.-W., Jan Y.-K. (2021). Estimation of Various Walking Intensities Based on Wearable Plantar Pressure Sensors Using Artificial Neural Networks. Sensors.

[35] Serj M.F., Lavi B., Hoff G., Valls D.P. (2018). A Deep Convolutional Neural Network for Lung Cancer Diagnostic. arXiv.

[36] Sriramakrishnan P., Kalaiselvi T., Padmapriya S.T., Shanthi N., Ramkumar S., Kalaichelvi N. (2019). An Medical Image File Formats and Digital Image Conversion. Int. J. Eng. Adv. Technol..

[37] Ujgare N.S., Baviskar S.P. (2013). Conversion of DICOM Image in to JPEG, BMP and PNG Image Format. Int. J. Comput. Appl..

[38] Oladiran O., Gichoya J., Purkayastha S. (2017). Conversion of JPG Image into DICOM Image Format with One Click Tagging. Proceedings of the Lecture Notes in Computer Science.

[39] Wiggins R.H., Davidson H.C., Harnsberger H.R., Lauman J.R., Goede P.A. (2001). Image File Formats: Past, Present, and Future1. RadioGraphics.

